# β-Glucan phosphorylases in carbohydrate synthesis

**DOI:** 10.1007/s00253-021-11320-z

**Published:** 2021-05-10

**Authors:** Zorica Ubiparip, Marc De Doncker, Koen Beerens, Jorick Franceus, Tom Desmet

**Affiliations:** Centre for Synthetic Biology (CSB), Department of Biotechnology, Ghent University, Coupure Links 653, 9000 Ghent, Belgium

**Keywords:** β-Glucan phosphorylases, Carbohydrate synthesis, β-Glucans

## Abstract

**Abstract:**

β-Glucan phosphorylases are carbohydrate-active enzymes that catalyze the reversible degradation of β-linked glucose polymers, with outstanding potential for the biocatalytic bottom-up synthesis of β-glucans as major bioactive compounds. Their preference for sugar phosphates (rather than nucleotide sugars) as donor substrates further underlines their significance for the carbohydrate industry. Presently, they are classified in the glycoside hydrolase families 94, 149, and 161 (www.cazy.org). Since the discovery of β-1,3-oligoglucan phosphorylase in 1963, several other specificities have been reported that differ in linkage type and/or degree of polymerization. Here, we present an overview of the progress that has been made in our understanding of β-glucan and associated β-glucobiose phosphorylases, with a special focus on their application in the synthesis of carbohydrates and related molecules.

**Key points:**

• *Discovery, characteristics, and applications of β-glucan phosphorylases.*

• *β-Glucan phosphorylases in the production of functional carbohydrates.*

## Introduction

**Fig. 1:**
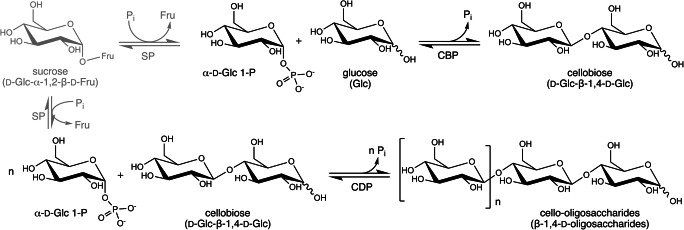
Example of the coupled reaction by β-glucobiose (cellobiose) phosphorylase, β-glucan (cellodextrin) phosphorylase, and sucrose phosphorylase for cellodextrin synthesis. Adapted from Ubiparip et al. (2020)

β-Glucan phosphorylases (β-GPs) are carbohydrate-active enzymes that catalyze the degradation of β-glucans (β-Gs) with the use of inorganic phosphate, yielding α-d-glucose 1-phosphate (α-G1P) and a shorter carbohydrate chain as products (Fig. 1). Because of the high energy content of the glucosyl phosphate, the reaction is readily reversible and can be used for the synthetic purposes of β-glucans in vitro. In that case, the donor substrate α-G1P serves as a shorter and, more importantly, cheaper version of the UDP-Glc required by the corresponding “Leloir” glycosyltransferases (e.g., β-1,3-glucan synthase) and can be conveniently prepared through the phosphorolysis of cheap and abundant resources like starch or sucrose (De Winter et al. 2011). Interestingly, the donor can even be generated in situ by coupling the action of starch or sucrose phosphorylases and β-GPs in a one-pot reaction (Fig. 1) (Abe et al. 2015; Müller et al. 2017b; Zhong and Nidetzky 2019). In addition to sucrose phosphorylase, β-GPs can simultaneously or consecutively be coupled with β-glucobiose phosphorylases, such as cellobiose or laminaribiose phosphorylase, to enable β-glucan production starting from glucose as an inexpensive acceptor (Fig. 1) (Abe et al. 2015; Müller et al. 2017b; Zhong and Nidetzky 2019). Finally, β-glucan and β-glucobiose phosphorylases can be used for the enzymatic glycosylation of non-carbohydrate acceptors (e.g., drugs) to increase their activity, pharmacokinetic properties, and solubility, or to reduce their toxicity (Desmet et al. 2012; De Winter et al. 2015a; De Winter et al. 2015b).

**Fig. 2:**
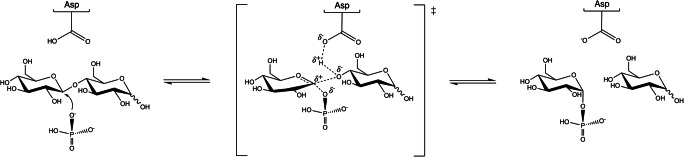
Simplified scheme of the single displacement mechanism of β-glucan phosphorylases. Phosphate performs a nucleophilic attack on the anomeric carbon, while a catalytic acid activates the leaving group by accepting a proton

The majority of the known β-GPs are classified in glycoside hydrolase family 94 (GH94), which includes five different phosphorylase specificities (Table 1), as well as cyclic β-1,2-glucan synthase, which has both glycosyl transferase 84 (GT84) and GH94 domains (Lombard et al. 2014; Kitaoka 2015) (www.cazy.org). Recently, glycoside hydrolase families 149 (GH149) and 161 (GH161) have been established (Table 1). They comprise four characterized enzymes that act on β-1,3-linked oligo and polysaccharides (Kuhaudomlarp et al. 2018; Kuhaudomlarp et al. 2019b) (Table 2). Members of all three families utilize the same single displacement mechanism that results in inversion of the anomeric configuration (Fig. 2).

**Table 1:** Known β-glucan and β-glucobiose phosphorylase specificities

Phosphorylase specificity	Abbreviation	Enzyme family	Linkage	DP^a^	EC	Reference
β-1,2-Oligoglucan	SOGP	GH94	β-1,2	2–25	2.4.1.333	Nakajima et al. (2014)
Laminaribiose	LBP		β-1,3	2	2.4.1.31	Nihira et al. (2012)
Cellobiose	CBP		β-1,4	2	2.4.1.20	Alexander (1968)
Cellodextrin	CDP		β-1,4	3–10	2.4.1.49	Sheth and Alexander (1969)
β-1,3-Oligoglucan	BOP	GH94, GH149	β-1,3	2–30	2.4.1.30	Maréchal and Goldemberg (1963); Maréchal (1967); Ogawa et al. (2014); Kuhaudomlarp et al. (2018)
β-1,3-Glucan	BGP	GH161	β-1,3	3–34	2.4.1.97	Nakai et al. (2013); Yamamoto et al. (2013); Kuhaudomlarp et al. (2019a)

^a^Approximate DP (degree of polymerization) range of synthesized carbohydrates

**Table 2:** Overview of the characterized β-glucan and β-glucobiose phosphorylases and their features

Enzyme family	Species	T_opt_ (°C)	pH_opt_	Substrate^a^	*K*_*m*_ (mM)^b^	*k*_cat_ (s^−1^)^b^	PDB entry	Reference
CDPs								
GH94	*Clostridium stercorarium*	65	6–7	Glucose Cellobiose	– 0.4	– 18	–	Reichenbecher et al. (1997); Tran et al. (2011)
	*Clostridium thermocellum* YM4	60	7.5	Cellobiose (Cel_2_) Cel_3_ Cel_4_	0.9 1.7 2.6	10.1 13.9 16.2	5NZ7, 5NZ8	Krishnareddy et al. (2002); O’Neill et al. (2017)
	*Clostridium cellulosi*	45–60	7	Glucose, cellobiose, *p*-nitrophenyl β-cellobioside	–	–	–	Zhong et al. (2019); Zhong et al. (2020a)
	*Ruminococcus albus*	50	6	Cellobiose (Cel_2_) Cel_3_ Cel_4_ Cel_5_ Cel_6_ Sophorose Laminaribiose Xylobiose Mannobiose Cellobiitol	13.2 5 4 2.7 3.2 343 119 50.9 65 73.7	47.1 43.7 37.8 28.9 18.2 14.2 33.3 15.4 41.3 3.3	–	Sawano et al. (2013)
CBPs								
GH94	*Clostridium thermocellum*	–	6.5 (d-Xylose) 7.5 (2-Deoxy-d-glucose)	6-Deoxy-d-glucose Xylose 2-Deoxy-d-glucose Glucosamine Mannose Arabinose l-Fucose α-d-Glucose 1-P	9.2 35 73 9.5 85 240 160 2.1	–	3QDE	Alexander (1968); Bianchetti et al. (2011); De Winter et al. (2015b)
				l-Galactose, altrose, pentanol, hexanol, 2-hexanol, cyclohexanol, heptanol, octanol, 2-octanol, nonanol, decanol, dodecanol, β-citronellol, geraniol, anisyl alcohol, cinnamyl alcohol, pyrogallol, 2-phenylethanol, vanillyl alcohol, vanillin, hydroquinone	–	–		
	*Clostridium thermocellum* ATCC 27405	60	6–8	Mannose Xylose Glucosamine 2-Deoxy-d-glucose α-d-Glucose 1-P	170 40 10 250 8	–	–	Tanaka et al. (1995)
	*Clostridium stercorarium*	65	6–7	Glucose	–	–	–	Reichenbecher et al. (1997)
	*Cellvibrio gilvus*	–	7.6	Glucose Mannose 2-Deoxy-d-glucose Glucosamine 6-Deoxy-d-glucose Xylose	2.1 115 168 13 24 84	–	2CQS, 2CQT	Kitaoka et al. (1992); Percy et al. (1998); Hidaka et al. (2004); Hidaka et al. (2006)
				Melibiose, gentiobiose, isomaltose, glucuronamide	–			
	*Cellulomonas uda*	–	–	Glucose β-d-Glucose Mannose 2-Deoxy-d-glucose 2-Deoxy-2-fluoro-d-glucose Glucosamine 3-Deoxy-d-glucose 3-Deoxy-3-fluoro-d-glucose 6-Deoxy-d-glucose 6-Deoxy-6-fluoro-d-glucose Xylose α-d-Glucose 1-P	2.3 4.7 27.3 24.3 17.2 10.8 4 – 10.2 7.4 15.9 2.1	44.2 47.6 6.6 11.6 8 5.6 0.1 – 55.3 44.6 8.7 –	3S4A, 3S4B, 3RRS, 3RSY, 3ACS, 3AFJ, 3ACT, 3QG0, 3QFY, 3QFZ	Van Hoorebeke et al. (2010); Bianchetti et al. (2011); Fushinobu et al. (2011)
	*Prevotella ruminicola* (crude cell extract)	–	–	–	–	–	–	Lou et al. (1996)
	*Ruminococcus albus* NE1	50	6.2	Glucose Mannose 2-Deoxy-d-glucose Glucosamine Xylose 6-Deoxy-d-glucose 1,5-Anhydro-d-glucitol Gentiobiose	1.5 23.5 60.2 13.3 25.5 9.8 9.5 10.8	92.3 2.8 21.5 9.6 16.5 124 2.8 4	–	Hamura et al. (2012)
	*Thermotoga neapolitana*	85	5	Glucose	–	–	–	Yernool et al. (2000)
	*Thermotoga maritima* MSB8	80	6.2	Glucose Mannose Glucosamine Xylose 2-Deoxy-d-glucose 6-Deoxy-d-glucose Methyl-β-d-glucoside	0.7 67 5.7 14 47 4.1 135	8 4.4 5.2 40 16 17 6.5	–	Rajashekhara et al. (2002)
LBPs								
GH94	*Acholeplasma laidlawii* PG-8A	40	6	Glucose 2-Deoxy-d-glucose	0.4 0.7	1.4 1	–	Nihira et al. (2012)
				Xylose, d-glucuronic acid, 1,5-anhydro-d-glucitol, mannose	–	–		
	*Paenibacillus* sp. YM1	55	6.8–7	Glucose	6	15	–	Kitaoka et al. (2012); Kuhaudomlarp et al. (2019c)
				Mannose, allose, galactose, l-idose, α-methylglucoside, β-methylglucoside, 1,5-anhydroglucitol, 2-deoxy-d-glucose, glucosamine, N-acetylglucosamine, 4-deoxy-d-glucose, 6-deoxy-d-glucose, xylose, lyxose, ribose, l-arabinose, fructose, l-sorbose, phenyl β-glucoside, cellobiose, laminaribiose, sucrose	–	–	–	
BOPs								
GH94	*Ochromonas danica*	25–30	5.5	Laminaribiose (Lam_2_), Lam_3_, Lam_4_, Lam_5_, Lam_6_, laminarin, cellotriose (Cel_3_), Cel_4_, Cel_5_, sophorose, methyl β-d-glucopyranoside, *p*-nitrophenyl β-d-glucopyranoside	–	–	–	Yamamoto et al. (2013)
	*Poterioochromonas malhamensis*	22.5	5.5	Laminarin, chrysolaminarin, cellobiose, glucose, maltose, β-methylglucoside	–	–	–	Kauss and Kriebitzsch (1969); Albrecht and Kauss (1971)
GH149	*Euglena gracilis*	30	6.3–6.9	Glucose Laminaribiose (Lam_2_) Lam_3_ Lam_4_ Lam_5_ Lam_6_	0.6 0.7 1.3 1.4 2.3 2.9	1.1 1.1 1.1 1.1 1.1 1.1	–	Maréchal and Goldemberg (1963); Goldemberg et al. (1966); Kitaoka et al. (1993); Kitaoka et al. (2012); Kuhaudomlarp et al. (2018)
				Laminarin, allose, mannose, galactose, l-idose, β-methyl-d-glucoside, 1,5-anhydro-d-glucitol, 2-deoxy-d-glucose, glucosamine, N-acetylglucosamine, d- or l-xylose, sophorose, cellobiose, gentiobiose, phenyl-β-d-glucoside, *O*-nitrophenyl-β-d-glucoside, *m*-nitrophenyl-β-d-glucoside, *p*-nitrophenyl-β-d-glucoside, salicin, maltose, paramylon treated with KOH, α-methylglucoside, β-methylglucoside, 6-deoxyglucose, lyxose, ribose, l-arabinose, fructose, l-sorbose, sucrose	–	–	–	
	Pro_7066 (bacterial, from metagenomic library)	–	–	Glucose Laminaribiose (Lam_2_) Lam_3_ Lam_4_ Lam_5_ Lam_6_	0.3 0.2 0.4 0.4 0.3 0.3	1.7 1.5 1.5 1.4 1.3 1.1	6HQ6, 6HQ8	Kuhaudomlarp et al. (2018); Kuhaudomlarp et al. (2019b)
BGPs								
GH161	*Thermosipho africanus* TCF52B	75	7.5	Glucose Cellobiose (Cel_2_) Cel_3_ Cel_4_ Cel_5_ Xylose α-d-Glucose 1-P	14.3 7.5 4 3.7 2.6 25.7 1.6	30.3 361 603 612 527 1.4 7.6	–	Wu et al. (2017); Kuhaudomlarp et al. (2019a)
	*Paenibacillus polymyxa* ATCC 842	–	–	Laminaribiose (Lam_2_) Lam_3_ Lam_4_ Lam_5_ Lam_6_	1.6 1 1.8 1.6 2.3	32 33 33 27 30	–	Kuhaudomlarp et al. (2019a)
SOGPs								
GH94	*(Lachno)Clostridium phytofermentans* ISDg	40	7	Sophorose (Sop_2_) Sop_3_ Sop_4_ Sop_5_ α-d-Glucose 1-P	40 55 46 – 28	5.5 2.8 2.2 – 2.6	5H40	Nakajima et al. (2017)
	*Listeria innocua* Clip11262	37–45	7.5–8	Sophorose (Sop_2_) Sop_3_ Sop_4_ Laminaribiose Glucose α-d-Glucose 1-P	8.5 6 6.8 – – 1.2	97 110 90 – – 43	–	Nakajima et al. (2014)

^a^Unless stated otherwise, all acceptors are in d- (dextro) form

^b^Kinetic properties are considered in synthetic direction; standard deviation is not included

– not available

In 2013, Nakai et al. summarized the general use of phosphorylases for oligosaccharide synthesis (Nakai et al. 2013). The last comprehensive review on the diversity of these enzymes and their applications was published in 2015 by Kitaoka (Kitaoka 2015). Since then, the collection of novel β-glucan phosphorylases has expanded significantly, and numerous authors described their use in the synthesis of functional oligosaccharides. To encourage future discoveries involving β-GPs and to emphasize their potential for the easy, sustainable, and cost-friendly synthesis of valuable carbohydrates, we present a comprehensive overview of their features and current applications.

## Discovery of β-glucan and β-glucobiose phosphorylases

The earliest work on potential sources, isolation, purification, and characterization of β-glucan phosphorylases was carried out in the 1960s and 70s. The first β-GP was isolated from the versatile phototrophic protist *Euglena gracilis* (Maréchal and Goldemberg 1963; Maréchal 1967), followed by the extraction and partial purification of another homolog from the unicellular algae *Poterioochromonas malhamensis* (Table 2) (Kauss and Kriebitzsch 1969). Both enzymes are active on β-1,3-oligoglucans and are, together with the enzyme from *Paenibacillus polymyxa*, the only known β-1,3-oligoglucan phosphorylases that cannot use glucose as acceptor (Table 2). Today, three different phosphorylase specificities have been described that involve the disaccharide laminaribiose or β-1,3-glucans, i.e., laminaribiose phosphorylase (LBP), β-1,3-oligoglucan phosphorylase (BOP), and β-1,3-polyglucan or laminarin phosphorylase (BGP) (Table 1). Although all can degrade the characteristic β-1,3-glycosidic linkage, they exhibit a different preference for the chain length of their substrate (Nakai et al. 2013; Yamamoto et al. 2013). In the CAZy-classification (Lombard et al. 2014) (www.cazy.org), these enzymes are organized into three distinctive families (GH94, GH149, and GH161), which comprise eight described enzymes (Table 2). The inability of some BOPs and BGPs to use glucose as an acceptor for β-G synthesis can be overcome through coupled reactions with laminaribiose phosphorylases. Two LBPs have been characterized to date. One originates from *Acholeplasma laidlawii* (Nihira et al. 2012) and the other from *Paenibacillus* sp. (Kitaoka et al. 2012; Kuhaudomlarp et al. 2019c), with the former displaying a far higher affinity for glucose (Table 2). Most β-1,3-GPs show a fairly broad acceptor specificity, which could diversify their application potential in the carbohydrate industry (Table 2). Their optimal operational temperature ranges from 25 °C for β-GP from *Ochromonas danica* (*Od*BGP), up to 75 °C for the homolog from *Thermosipho africanus* (*Ta*BGP) (Table 2). *Ta*BGP is the only known and well-described thermostable β-1,3-glucan phosphorylase (Kuhaudomlarp et al. 2019a). Interestingly, the enzyme was initially believed to synthesize β-1,4-linkages to yield cellodextrins, but that was later found to be a mistake (Wu et al. 2017). Indeed, structural analysis of the oligosaccharide products revealed that *Ta*BGP is specific to β-1,3-oligosaccharides, and the enzyme was subsequently reclassified into a newly established GH161 family (Kuhaudomlarp et al. 2019a).

Shortly after the discovery of the first β-GP from *E. gracilis*, a related enzyme that catalyzes the reversible phosphorolysis of cellobiose into α-G1P and glucose was identified (Fig. 1; Table 2) (Alexander 1968). The enzyme was isolated from the cellulolytic bacterium *Clostridium thermocellum* and designated as cellobiose phosphorylase (CBP). This specificity remains the most studied one in family GH94, with numerous variants, crystal structures, production processes, and engineering studies reported in the scientific literature (Kitaoka 2015). The first cellodextrin phosphorylase (CDP) was discovered a year later in another member of the *Clostridium* genus (Fig. 1; Table 2) (Sheth and Alexander 1969). CDPs can be used to synthesize longer β-1,4-oligosaccharides or cellodextrins, but cellobiose is typically the shortest carbohydrate that they can recognize as acceptor substrate. The majority of the known cellobiose and cellodextrin phosphorylases originate from the same genus, comprising mainly anaerobic cellulolytic bacteria where these enzymes play a role in a complex multi-enzymatic cluster that enables the utilization of cellulose as a carbon source (Table 2) (Liu et al. 2019). Concerning their biochemical features, most CBPs and CDPs are stable at high temperatures, and their pH optima are in line with those of other known phosphorylases, ranging between 6 and 8 (Table 2) (Ubiparip et al. 2018). Interestingly, both CBP and CDP have the broadest acceptor and donor substrate specificity of all GH94 enzymes, making them suitable candidates for the biocatalytic synthesis of diverse carbohydrates and related molecules (Table 2).

Finally, β-1,2-GPs (sophoro-oligosaccharide-GPs, SOGPs) have been reported, but these specificities are somewhat understudied (Table 2). Quite recently, Nakajima et al. identified the only two representatives known to date, originating from *Listeria innocua* (*Li*SOGP) (Nakajima et al. 2014) and *Lachnoclostridium phytofermentans* (*Lp*SOGP) (Nakajima et al. 2017). Both enzymes are classified in GH94 and have a relatively narrow substrate specificity with low affinities for natural β-1,2-oligosaccharide acceptors (*K*_*m*_ ≥ 6 mM) (Table 2). In contrast to the β-1,3- and β-1,4-GPs, no thermostable β-1,2-glucan phosphorylase has been identified so far (Table 2).

While linear β-1,6-glucans (from *Umbilicaria pustulata*) (Barreto-Bergter and Gorin 1983) and their *O*-acetylated (from *Gyrophora esculenta*, *Lasallia papulosa*, *Sticta* sp.) (Shibata et al. 1968; Da Silva et al. 1993), and malonic ester forms (from *Penicillium luteum*) (Anderson et al. 1939) can be found in nature, the corresponding β-glucan phosphorylases have yet to be discovered.

## β-Glucan phosphorylases in β-glucan synthesis

**Fig. 3:**
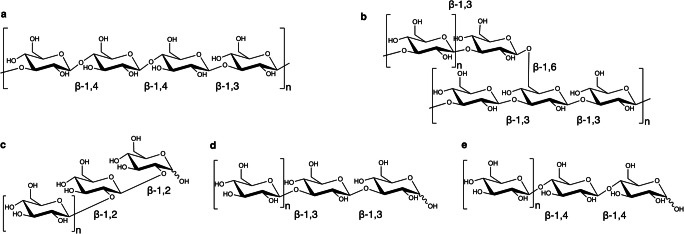
Characteristic structures of several β-glucans. **a** β-Glucans originating from cereals contain a mixture of β-1,4 and β-1,3 linkages, typically β-1,3-linked cellotriosyl or tetraosyl blocks, **b** β-glucans originating from fungi contain a characteristic linear β-1,3 backbone, branched with β-1,6 linkages, **c** the structure of linear β-1,2-, **d** β-1,3-, and **e** β-1,4-oligosaccharides, which can respectively be synthesized by SOGP, BGP/BOP, and CDP

β-Glucans are polysaccharides consisting of β-d-glucose monomers linked by β-1,2, β-1,3, β-1,4, or β-1,6 glycosidic linkage that show a diverse range of physicochemical properties depending on the source, type of glycosidic bond, and the length of the polysaccharide chain (Fig. 3).

Most of the β-glucans produced on a commercial scale today are extracted from the cell walls of yeasts, fungi, and plants, although some are synthesized by fermentation (Zhu et al. 2016; Liang et al. 2018). The purified β-Gs are typically obtained by acidic hydrolysis steps, followed by selective precipitation using organic solvents (Shi 2016; Zhu et al. 2016). The extraction process and biological origin of β-Gs lead to significant variations in their physicochemical and functional properties, including their branching pattern, molecular weight distribution, viscosity, and concentration in the biological matrix (Zhu et al. 2016).

Enzymatic production processes would likely provide tighter control over the product composition while also eliminating the need for organic solvents. Although certain glycosyltransferases are specialized in the synthesis of β-Gs (Douglas 2001), the high cost of their nucleotide-activated donor sugars is a serious limitation for their commercial exploitation (Mikkola 2020). Due to their ability to use the easily accessible donor α-G1P, β-glucan phosphorylases seem better suited for cost-effective industrial use.

### β-1,2-Glucan phosphorylases

In nature, β-1,2-glucans are produced by bacteria and play an important role in the invasion and immunomodulation of infected mammalian or plant cells (Zhang et al. 2016). Furthermore, the β-1,2-linked disaccharide sophorose is a potent inducer of cellulase gene expression in the host *Trichoderma reesei* (Sternberg and Mandels 1979). Sophorose can be easily isolated and purified as a side product during commercial sophorolipid production by the yeast *Candida bombicola* (Claus and Van Bogaert 2017). These sophorolipids can serve as biosurfactants in, for instance, biological detergents and are known to have antibacterial, antifungal, spermicidal, and virucidal activities (Geys et al. 2014).

Two authors described the synthesis of β-1,2-glucans on a small scale using the β-1,2-oligoglucan phosphorylase from *Listeria innocua* (*Li*SOGP) (Nakajima et al. 2014). In comparison with the enzyme from *Lachnoclostridium phytofermentans* (*Lp*SOGP), *Li*SOGP has a higher affinity for β-1,2-oligosaccharides as acceptors and might be more suitable for β-1,2-G synthesis, though its catalytic efficiency is somewhat lower (Table 2). For example, 167 g of β-1,2-G could be obtained through a coupled reaction with sucrose phosphorylase from *Bifidobacterium longum* in a 1 L reactor starting from 1 M sucrose, 0.5 M glucose, and 0.1 M inorganic phosphate (Abe et al. 2015). The current bottleneck in β-1,2-G production is the low affinity of β-1,2-GPs for glucose. Indeed, the enzyme has a strong preference for sophorose, but this substrate is too expensive to justify its use in large-scale productions (Table 2) (Nakajima et al. 2014; Nakajima et al. 2017). To overcome those drawbacks, a three-step process was designed (Kobayashi et al. 2019). First, β-1,2-gluco-oligosaccharides were generated from a very small amount of sophorose (20 μg) by the combined action of sucrose phosphorylase, β-1,2-glucanase, and *Li*SOGP. This reaction was sequentially repeated two more times in increasing volumes, each time using the product from the previous reaction as an acceptor substrate. After the third and final cycle, 140 g/L of β-1,2-glucan was obtained on a 1 L scale (Kobayashi et al. 2019). The discovery or engineering of SOGPs with higher thermostability and affinity towards glucose, as well as additional synthesis of β-1,2-glucans, is expected to facilitate investigation of their physiological functions, physicochemical properties, and their use as functional carbohydrates.

### β-1,3-Glucan phosphorylases

β-1,3-Glucans draw considerable attention for their proven beneficial effects on immunomodulation, cholesterol levels, and glycemic control, and their use as additives in food or moisturizing personal care products (Rahar et al. 2011; Nie et al. 2018; Vetvicka et al. 2019). In the USA, the Food and Drug Administration (FDA, 1997, 2003) has allowed a heart health claim for products containing β-Gs from oat or barley, typically comprised of mixed β-1,3:1,4-linkages (Fig. 3). The EU approved the health claim related to the regular consumption of these β-Gs for the maintenance of normal blood cholesterol levels and the reduction of blood glucose increase after the meal (Sibakov et al. 2012). Currently, there are various commercial β-glucan-containing food products already on the market, such as Glucagel (Morgan and Ofman 1998), Oatrim (Greenway et al. 2007), and Viscofiber (Colla et al. 2018), where β-Gs from oat and barley serve as functional additives, fat replacers, or aid in weight loss. Moreover, the disaccharide laminaribiose has prebiotic properties (Kumar et al. 2020) and is a powerful germination agent (Jamois et al. 2008).

The first enzymatic production process of laminari-oligosaccharides from glucose and α-G1P was reported in 1991, more than 20 years after the enzyme’s discovery, using *Eg*BOP crude cell extract (Kitaoka et al. 1991b). The authors showed that the concentration of glucose could be used to control the product’s degree of polymerization (DP) since the average DP of laminari-oligosaccharides was 1.8 with 100 mM glucose, while the reaction with 5 mM glucose yielded a product with an average DP of 8.4 (Kitaoka et al. 1991b). In 1992, a first patent was submitted describing a similar process to obtain laminari-oligosaccharides of DP2-4 using *Euglena* cell extracts (Ito et al. 1994), followed by another that made use of a thermotolerant laminaribiose phosphorylase from the *Bacillus* genus (Mitsuyoshi et al. 2001). More recently, Yamamoto and co-workers reported the production and characterization of BGP from *Ochromonas danica* (Yamamoto et al. 2013). The enzyme was also biochemically characterized and could phosphorolyze β-1,3-linked oligo- and polysaccharides but not the disaccharide laminaribiose (Table 2). Similarly, glucose does not serve as an acceptor in the synthetic reaction (Yamamoto et al. 2013). The results of this research led to a patent describing the operating conditions for the manufacture of β-1,3-glucans of different degrees of polymerization (Isono et al. 2013). In 2017, it was reported that laminaribiose could be generated from sucrose and glucose, using *Eg*BOP and sucrose phosphorylase (Müller et al. 2017b). The covalent immobilization of cell extract of *Euglena gracilis* on Sepabeads EC-EP/S resulted in a high retained activity of *Eg*BOP (65%) and 14 g/L of laminaribiose (Müller et al. 2017b). The method was further improved by combined immobilization and entrapment in chitosan, allowing complete preservation of the enzymatic activity after 12 reuses (Müller et al. 2017a; Müller et al. 2017b). Coupling of this hybrid-immobilization with reaction-integrated laminaribiose extraction by adsorption on zeolites yielded 32 g/L of laminaribiose (Müller et al. 2017a). Another system made use of a packed bed reactor to immobilize *Eg*BOP and sucrose phosphorylase, enabling the production of 0.4 g/(L·h) of laminaribiose (Abi et al. 2018). The system was operationally stable during 10 days of processing, and both enzymes exhibited a half-life time of more than 9 days (Abi et al. 2018). Combined with integrated downstream processing by zeolites, it led to the production of over 0.5 g of laminaribiose per 1 g of sugar used as a substrate (Abi et al. 2019). A simplified preparation method of linear water-insoluble β-1,3-glucans of DP30, using sucrose phosphorylase and *Eg*BOP cell extract, led to a reaction system that requires only 0.1 mM glucose, 200 mM sucrose, and 20 mM phosphate as substrates (Ogawa et al. 2014). Thanks to its low solubility in water, approximately 1 mg/mL of glucan was conveniently isolated by precipitation (Ogawa et al. 2014). Finally, the production of *Eg*BOP in bioreactor has been optimized as well, resulting in around 2-fold higher activity compared to the flask cultivation, thus facilitating future applications of this enzyme in laminaribiose and β-Gs synthesis (Abi et al. 2017).

### β-1,4-Glucan phosphorylases

Cellulose or insoluble β-1,4-glucan is the major polysaccharide found in nature with an essential role as a structural polymer and material, while soluble cellodextrins of DP ≤ 6 have promising applications as nutritional ingredients, excipients in medicines, and texturizers in cosmetics (Sakamoto et al. 2017; Putta et al. 2018; Brucher and Häßler 2019; Zhong and Nidetzky 2019). Such soluble cellodextrins were proven to be useful dietary fibers and prebiotics (Otsuka et al. 2004; Pokusaeva et al. 2011) that stimulate the growth of a number of healthy human gut bacteria more efficiently than inulin, trans-galacto-oligosaccharides, and cellobiose alone (Zhong et al. 2020b).

Well-researched enzymatic pathways for cellulose synthesis mostly involved trans-glycosylation by glycoside hydrolases and synthesis by glycosynthases (Kitaoka 2015; O’Neill and Field 2015; Nidetzky and Zhong 2020). Samain and co-authors described the synthesis of crystalline cellulose II by CDP from *Clostridium thermocellum* using 2.5 mM cellobiose and 100 mM α-G1P (Samain et al. 1995). Years later, an alternative route starting from 50 mM glucose and 200 mM α-G1P was reported (Hiraishi et al. 2009). The average DP of cellulose in both studies ranged from 8 to 10, suggesting that CDP cannot elongate precipitated cellodextrin chains. Others later succeeded in producing cellulose with increased chain length (DP ≤ 14) by using very low concentrations of cellobiose (0.2 mM) (Petrović et al. 2015). Nonetheless, several authors have described the use of cellulases that, under specific conditions, can elongate chains to DP ≥ 100 and, therefore, could represent better biocatalysts for cellulose synthesis than CDP (Kobayashi et al. 1991; Egusa et al. 2007; Egusa et al. 2009; Egusa et al. 2010).

Nippon Petrochemicals and the National Food Research Institute in Japan were the first to patent the cellobiose production process (Kitaoka et al. 1991a). It was synthesized from 200 mM sucrose by the combined one-pot action of glucose isomerase and sucrose and cellobiose phosphorylase resulting in a yield of around 70% (relative to the concentration of donor substrate) (Kitaoka et al. 1991a). The synthesis of cellobiose from starch in a two-step reaction using α-glucan-phosphorylase from a rabbit muscle and cellobiose phosphorylase from *Cellvibrio gilvus* resulted in a relatively low yield of 24% (Suzuki et al. 2009). Pfeifer & Langen (Germany), one of the largest sugar manufacturers in Europe, developed a process to synthesize cellobiose from 750 mM sucrose in a one-pot reaction using sucrose phosphorylase from *Bifidobacterium adolescentis* (*Ba*SP) and an engineered CBP variant from *Cellulomonas uda* (*Cu*CBP) that resulted in 70% yield (Brucher and Häßler 2019). The cellobiose was subsequently purified by ultrafiltration for the separation and recycling of the enzymes and electrodialysis to recover phosphate and α-G1P, and finally, pure cellobiose was obtained by crystallization. Moreover, upscaling of the process was initiated by Savanna Ingredients GmbH (Germany) to about 100 tonnes per year (Brucher and Häßler 2019). Both the production process (Koch et al. 2016) and the *Cu*CBP variant (Koch et al. 2017) have been protected by a patent. Despite its promising applications as prebiotic and texturizer in food and feed products, cellobiose still requires approval as “Novel Food” by EFSA, the European Food Safety Authority (Brucher and Häßler 2019).

Several studies reported CDP catalyzed synthesis of soluble cello-oligosaccharides at the milligram scale. However, these studies were not envisaged for the efficient cellodextrin synthesis in terms of yields or high product concentrations (Samain et al. 1995; Zhang and Lynd 2006; Nakai et al. 2010). Recently, there has been an increasing interest in tailoring the bottom-up synthesis of soluble cellodextrins using CDPs as a cost-effective and ecologically friendly tool (Nidetzky and Zhong 2020). Zhong and co-authors obtained 36 g/L of soluble cellodextrins (DP3-6) from acceptor glucose using CDP from *Clostridium cellulosi* coupled with *Cu*CBP (Zhong et al. 2019). Moreover, a three-enzyme glycoside phosphorylase cascade was developed by introducing the *Ba*SP for in situ generation of α-G1P, which lead to 40 g/L of soluble cellodextrins produced (Zhong and Nidetzky 2019). Finally, an efficient synthesis of 93 g/L of soluble cellodextrins was demonstrated. The final product consisted of DP3, DP4, DP5, and DP6 with a distribution of 33, 34, 24, and 9 wt%, respectively, a purity of over 95%, and a yield of 88% (Zhong et al. 2020b). We previously described the synthesis of predominantly cellotriose from both glucose and cellobiose by using a cellobiose phosphorylase variant (Ubiparip et al. 2020). Facilitated synthesis of cellodextrins from glucose will certainly unlock further application studies, especially related to the use of these carbohydrates as food and feed additives.

It should be mentioned that β-glucan phosphorylases could also be employed to degrade β-glucans by phosphorolysis and produce α-G1P using inorganic phosphate as co-substrate, but their role in carbohydrate synthesis is industrially more valuable. Though α-G1P alone can be used as a substitute for inorganic phosphate in parenteral nutrition and as a supplement in medical conditions that involve phosphate deficiency, there is a limited need for it as a fine chemical (Ronchera-Oms et al. 1995; Luley-Goedl and Nidetzky 2010). Moreover, other enzymes such as α-glucan phosphorylases can be used for the same purpose and are active on cheaper and easily accessible substrates such as starch and maltodextrins (Ubiparip et al. 2018). As described, sucrose phosphorylase was already successfully exploited in various coupled reactions to produce α-G1P in situ, which subsequently served as a substrate for β-glucan synthesis (Abe et al. 2015; Müller et al. 2017b; Zhong and Nidetzky 2019).

## β-Glucan and β-glucobiose phosphorylases as promiscuous biocatalysts

Most (β-glucan) phosphorylases show promiscuity towards various alternative donors and/or acceptors (Table 2) (Singh et al. 2020). For example, the cellodextrin phosphorylase from *Clostridium thermocellum* was shown to successfully utilize anomeric phosphates of xylose (Shintate et al. 2003), galactose (Tran et al. 2012), and glucosamine (O’Neill et al. 2017), although only a single monomer could be added to the acceptor substrate in these cases (Singh et al. 2020). In turn, a variety of sugar phosphates (of glucose, galactose, glucosamine, and mannose) could be offered to the β-1,3-oligoglucan phosphorylase Pro_7066 for the synthesis of new-to-nature analogs of human milk oligosaccharides (HMO) (Singh et al. 2020). These studies highlighted the innate ability of β-glucan phosphorylases to synthesize smaller glycans (Singh et al. 2020). Although the kinetic efficiencies of these processes are frequently over 100-fold lower than those of their natural reaction, they enable single turnover that is not typical for the enzymes naturally prone to build carbohydrate polymers *(*Singh et al. 2020*)*.

Similar to CDP, the related cellobiose phosphorylases also have a broad acceptor and donor specificity (Table 2) and can be used in a number of alternative production processes that go beyond cellobiose synthesis. The first described CBP from *Clostridium thermocellum* was found to be active on a wide range of d- and l-glycosyl acceptors: d-glucose, 2-deoxyglucose, 6-deoxyglucose, d-glucosamine, d-mannose, d-altrose, l-galactose, l-fucose, d-arabinose, and d-xylose (Alexander 1968). Moreover, the enzyme successfully catalyzed the synthesis of a range of β-glucosides when adding solvents as reaction additives since disaccharide phosphorylases typically have a very low affinity for non-carbohydrate acceptors (De Winter et al. 2015b). Cellobiose phosphorylase from *Cellvibrio gilvus* was also successfully tested against 1,6-linked disaccharides (melibiose, gentiobiose, and isomaltose) as acceptors resulting in the corresponding β-1,4-capped trisaccharides (Percy et al. 1998).

Regarding β-1,3-glucan and glucobiose phosphorylases, BOP from *Euglena gracilis* and LBP from *Paenibacillus* sp. were shown to have the broadest acceptor specificity (Table 2). Laminaribiose phosphorylase from *Paenibacillus* sp. was able to use the alternative donor α-d-mannose 1-phosphate for the synthesis of mannosyl-β-1,3-glucose disaccharide (Kuhaudomlarp et al. 2019c). Moreover, the enzyme showed activity on various alternative acceptors, including mannose, methyl β-glucoside, 2-deoxyglucose, and 6-deoxyglucose, albeit with a 50- to 100-fold reduction in activity (Table 1) (Kitaoka et al. 2012). Laminarin phosphorylase from *Poterioochromonas malhamensis* could not use the alternative donor substrates glucose-1,6-diphosphate, fructose-l-phosphate, and fructose-1,6-diphosphate (Albrecht and Kauss 1971).

From all β-GPs reported so far, SOPGs have the narrowest substrate specificity, being highly specific to β-1,2-linked oligosaccharides (Table 2) (Nakajima et al. 2014; Nakajima et al. 2017). While α-G1P was the only sugar 1-phosphate used as a donor by *Li*SOGP, the enzyme showed some activity only on acceptors laminaribiose and glucose to, presumably, synthesize long-chained polysaccharides (Nakajima et al. 2014). Other monosaccharides such as mannose, galactose, xylose, fructose, N-acetylglucosamine, N-acetylgalactosamine, as well as a range of disaccharides (sucrose, maltose, lactose, cellobiose, etc.) were not utilized (Nakajima et al. 2014). Similarly, *Lp*SOGP showed only limited synthetic activity on laminaribiose, while no synthetic or phosphorolytic activity was observed on other tested substrates (Nakajima et al. 2017).

## Engineering of β-glucobiose and β-glucan phosphorylases

Relatively narrow substrate specificity prevents wide-range applications of disaccharide phosphorylases for glycoside synthesis (De Groeve et al. 2010a). Nonetheless, cellobiose and laminaribiose phosphorylase can be used for β-glucosylation or β-galactosylation of various molecules (Table 2) (De Groeve et al. 2010a; De Winter et al. 2015b). Lower affinity, acceptor and donor specificity, or specific activity of these enzymes related to non-natural substrates could be improved via enzyme engineering.

A decade ago, the acceptor specificity of CBP from *Cellulomonas uda* was successfully expanded through random mutagenesis. A double mutant (T508I/N667A) that no longer required a glucosyl acceptor with a free anomeric hydroxyl group and, hence showed some activity on cellobiose as acceptor was slightly improved (0.113 U/mg) by three additional mutations (N156D, N163D, and E649G) (De Groeve et al. 2010b). Most recently, it was determined that the mutant enzyme synthesizes mostly cellotriose with both glucose and cellobiose as acceptors (Ubiparip et al. 2020). The finding became particularly interesting since cellotriose was shown to be the most potent prebiotic among soluble cellodextrins (DP2-6) when tested against bifidobacteria of the human gut (Pokusaeva et al. 2011). The addition of M52R substitution improved the variant’s kinetic properties for the acceptor cellobiose and slightly increased the cellotriose yield (Ubiparip et al. 2020). This study was the first to demonstrate the possibility of controlled, bottom-up, enzymatic synthesis of cellodextrins with a specific degree of polymerization. In addition, the created *Cu*CBP variants showed activity on a range of alternative substrates (De Groeve et al. 2010b). The T508I and N667A mutations resulted in a 10-fold increase of lactose phosphorolysis by *Cu*CBP, which can be used to synthesize α-galactose 1-phosphate from the cheap and ample substrate lactose (De Groeve et al. 2010b). Quite recently, *Cu*CBP has been engineered to tolerate high substrate concentrations required by the industry in a coupled reaction process with *Cu*CBP and *Ba*SP. The variant contained eleven mutations (Q161M, R188K, D196N, A220L, L705T, Y164F, K283A, A512V, F164Y, S169V, and T788V) and showed greatly improved activity for the synthesis of cellobiose at higher substrate concentrations of up to 750 mM α-G1P and glucose, which in turn allowed efficient production of cellobiose in a one-pot reaction starting from 750 mM sucrose (Brucher and Häßler 2019). The Y47H substitution in the CBP from the yeast *Saccharophagus degradans* and the bacterium *Cellulomonas gilvus* significantly improved cellobiose consumption in the presence of xylose, a known inhibitor of the cellobiose phosphorylase, which is synthesized during enzymatic plant-biomass degradation (Chomvong et al. 2017). Finally, the stability of CBP from *Clostridium thermocellum* was increased by combining eight mutations (R48R, Q130H, K131Y, K142R, S411G, A423S, V526A, and A781K), therefore extending the enzyme’s inactivation halftime at 70 °C from 8 to 25 min (Ye et al. 2012).

Contrary to the engineering of *Cu*CBP, single-point mutagenesis of CDP from *Clostridium cellulosi* did not result in a change of the enzyme’s preference to synthesize specific cellodextrins, most probably due to its broader active site (Ubiparip et al. 2020). The creation of the C485A, Y648F, and Y648V mutants of the CDP from *Ruminococcus albus* resulted in the higher preference for d-glucosamine, more rapid synthesis of 4-*O*-β-d-glucopyranosyl-d-mannose, and synthetic activity on 4-*O*-β-d-glucopyranosyl-N-acetyl-d-glucosamine, respectively (Hamura et al. 2013). Since most CDPs have a low affinity for poorly soluble cellodextrin acceptors (Table 2), future engineering efforts might be directed towards decreasing the *K*_*m*_ for cellobiose or enabling the effective usage of more affordable acceptor glucose.

To this point, no authors described the engineering of the β-1,2- or β-1,3-(oligo)glucan phosphorylases. Recently solved crystal structures of both specificities are expected to encourage engineering efforts on these enzymes (Table 2) (Nakajima et al. 2017; Kuhaudomlarp et al. 2019b).

## Conclusions and perspectives

Since the discovery of the first β-glucan phosphorylase over half a century ago, several new specificities have been identified, including LBP, BGP, and SOGP during the last decade. Elucidation of the first crystal structures of CDP, BOP, and SOGP has recently also been achieved. The cumulative research interest in β-glucan and glucobiose phosphorylases has led to biosynthetic routes for laminaribiose, cellobiose, soluble cellodextrins, as well as β-1,2- and β-1,3-glucans. Increasingly, these enzymes are recognized as valuable biocatalysts for the production of such carbohydrates, which can be used as ingredients and additives in the food, feed, and cosmetic industry. A prime example is the development and scale-up of a process for the production of cellobiose by Pfeifer & Langen and Savanna Ingredients GmbH.

Although a number of β-glucan and glucobiose phosphorylases have been characterized so far, future studies could focus on the discovery of novel specificities such as β-1,6-glucan and β-1,6- or β-1,2-glucobiose phosphorylase. Undoubtedly, the biotechnological potential of these phosphorylases will continue to rise in the coming years. Enzyme engineering could lead to new and improved variants, as already demonstrated by the example of the cellobiose phosphorylase from *Cellulomonas uda*. The research focused on fine-tuning of operating conditions could result in industrially relevant and scalable production processes, which could, in turn, allow in-depth characterization of the properties and applications of the β-glucan products. Further investigations of the structure-function relationships of both β-glucan phosphorylases as well as the synthesized β-glucans will go hand in hand and lead to the upcoming commercialization of both β-GPs and β-Gs. Finally, these developments could result in the production of healthier carbohydrates (i.e., functional foods), now more relevant than ever due to the global rise in obesity and related health problems.
